# Modal Decomposition of Acoustic Emissions from Pencil-Lead Breaks in an Isotropic Thin Plate

**DOI:** 10.3390/s23041988

**Published:** 2023-02-10

**Authors:** Xinyue Yao, Benjamin Steven Vien, Nik Rajic, Cedric Rosalie, L. R. Francis Rose, Chris Davies, Wing Kong Chiu

**Affiliations:** 1 Department of Mechanical and Aerospace Engineering, Monash University, Wellington Rd., Clayton, VIC 3800, Australia; 2 Defence Science and Technology Group, 506 Lorimer St., Fishermans Bend, Port Melbourne, VIC 3207, Australia

**Keywords:** acoustic emission, wave propagation, Lamb wave, modal decomposition, structural health monitoring, spectrum analysis, 2D FFT, pencil lead break test

## Abstract

Acoustic emission (AE) testing and Lamb wave inspection techniques have been widely used in non-destructive testing and structural health monitoring. For thin plates, the AEs arising from structural defect development (e.g., fatigue crack propagation) propagate as Lamb waves, and Lamb wave modes can be used to provide important information about the growth and localisation of defects. However, few sensors can be used to achieve the in situ wavenumber–frequency modal decomposition of AEs. This study explores the ability of a new multi-element piezoelectric sensor array to decompose AEs excited by pencil lead breaks (PLBs) on a thin isotropic plate. In this study, AEs were generated by out-of-plane (transverse) and in-plane (longitudinal) PLBs applied at the edge of the plate, and waveforms were recorded by both the new sensor array and a commercial AE sensor. Finite element analysis (FEA) simulations of PLBs were also conducted and the results were compared with the experimental results. To identify the wave modes present, the longitudinal and transverse PLB test results recorded by the new sensor array at five different plate locations were compared with FEA simulations using the same arrangement. Two-dimensional fast Fourier Transforms were then applied to the AE wavefields. It was found that the AE modal composition was dependent on the orientation of the PLB direction. The results suggest that this new sensor array can be used to identify the AE wave modes excited by PLBs in both in-plane and out-of-plane directions.

## 1. Introduction

There has been a significant amount of research on the development of structural health monitoring techniques to detect fatigue crack growth [1,2,3] in aircraft components. The uncontrolled propagation of an internal fatigue crack in a structural component under loading can lead to catastrophic failure, so the early detection of fatigue cracks is desirable. Many non-destructive testing (NDT) methods have been applied to crack detection in metallic structures, including ultrasonics, eddy current, and X-ray computed tomography [4,5,6,7].

Acoustic emission (AE) is one of the few passive NDT techniques available for detecting, characterising, quantifying damages, and predicting system failure [8,9,10,11,12]. AE is defined as the rapid release of transient elastic waves from localised sources in solids [13]. Such emissions occur in metal, rock, cement, and composites [9,14,15,16,17]. Research has been undertaken on AEs generated during static tensile tests and fatigue tests of metallic material [8,10,18,19,20,21].

Generally, the analysis of AE signals mainly focuses on two aspects: hit-related signatures and waveform features. Hit-related signatures include parameters such as rise time, amplitude, and count rate, and waveform features include dominant frequencies, wave modes, and power spectral entropy [22]. In a study of hit-related features, Roberts and Talebzadeh conducted an experiment on compact steel specimens [10], finding that it was possible to use the linear correlation between the AE count rate and the crack propagation rate to predict the remaining life of a structure. A similar linear relationship has also been indicated in other studies [23,24]. As a result, the count rate can be used as an empirical tool for predicting the fatigue life remaining in specimens. In addition, many researchers have used cluster analysis, a data analysis method for classification of AE hits, to identify different types of AE sources [17,25,26].

A number of researchers have suggested that research should focus not only on the AE hit-related features, but also on the AE waveform features [20,27,28,29]. To capture AE waveforms, the most popular types of AE sensors used are bonded piezoelectric sensors and commercial AE sensors. Because the acoustic environment in most structural applications is noisy, it is customary to exclude from consideration emissions that do not exceed a predetermined signal threshold [30]. While excluding low-amplitude AE signals can lead to information loss, it is a simple and effective noise-mitigation technique and is widely used in practical implementations of AE testing.

In investigations of AE waveform features, researchers have focused on the dominant frequency contribution in the waveforms. In a study in which hits were synchronised with fatigue loading and clustered into nine groups according to the time domain signal and frequency spectrum of waveforms, Bhuiyan et al. found that these groups of hits had distinct peak frequencies for particular loads; the authors associated these with different sources [31]. Other researchers have also reported a correspondence between dominant frequencies and different AE sources [25,32,33,34], suggesting that the frequency composition of AE waveforms may be relatable to the type of source. However, other studies have also shown that the frequency spectrum of AE waveforms can vary depending on the type of sensor used [31]. As a result, it is important to consider the dynamic response characteristics of a sensor when undertaking such studies [35].

Due to the possible variability in the monitored frequencies, wave mode identification is potentially a better option for providing information about AE sources. The identification and analysis of contributing modes in an AE is a practice referred to as Modal Acoustic Emission (MAE), which previous studies have shown to be an effective method of AE source localisation and identification [36,37]. Hamstad [38] showed that in-plane pencil lead break (PLB) tests excite a distinct ratio of the flexural to the extensional mode when applied at the near top surface and near mid-plane locations in a plate. In Maslouhi [39], during an increase in the fatigue life percentage from 0% to 100%, the wavelet transform (WT) coefficient of a flexural wave mode increased first and then decreased, while that of an extensional wave mode kept decreasing.

Wave modes can be identified using a range of established methods, including time–frequency plots. Using the frequency–time domain WT method, experimentally obtained frequency–time domain contours are compared to contours determined theoretically from Lamb wave dispersion relations [29,38,39]. However, the frequency–time domain method only works when the WT coefficients of emitted wave modes are well-separated in time because, when the source–sensor distance is very short, the WT coefficients for different wave modes are close to each other and thus become hard to distinguish. Commercially available AE sensors only record waveforms at a single location, which limits the ability of these sensors to be used for modal separation if the source–sensor distance is relatively small.

A potentially more useful way of differentiating wave modes is to conduct a wavenumber–frequency decomposition by performing a two-dimensional fast Fourier transform (2D FFT). However, to determine the wavenumber of contributing wave modes, the AE must be resolved spatially. The Linear Array for Modal Decomposition and Analysis (LAMDA) is a new thin-film piezoelectric sensor array made of polyvinylidene fluoride (PVDF). This sensor array consists of 16 separate equidistant sensing elements, enabling the recording of 16 waveforms simultaneously, thus allowing the wavenumber–frequency decomposition of an AE [40,41]. LAMDA has been used to identify the emitted wave modes of ball-drop impacts [40], but the capabilities of LAMDA in identifying wave modes generated by PLB are yet to be investigated. PLB testing is a well-established method for generating AE sources [38] that are broadly representative of actual AE sources in components under stress and, therefore, are significantly more representative than a ball-drop impact. Consequently, an experimental assessment of the performance of LAMDA in the modal decomposition of PLB signals is considered an important preliminary step toward the eventual implementation of this sensor for the AE monitoring of components under loading.

Using the LAMDA sensor, a commercial AE sensor, and finite element analysis (FEA), this study investigates the capabilities of the LAMDA sensor in decomposing AE wave modes generated by PLBs applied to a thin metal plate. Two different orientations of the PLBs were used to create AE sources, specifically along the longitudinal and the transverse direction of the test plate. In the present work, a novel implementation of the conventional PLB is used, where a reference hole is drilled in the thickness direction of the plate into which the lead is inserted, in order to control the location and orientation of the PLB. These test configurations give rise to multiple Lamb wave modes in the test plate, which are identified through modal decomposition using LAMDA sensors.

## 2. Methods

### 2.1. PLB Experiments

**Figure 1:**
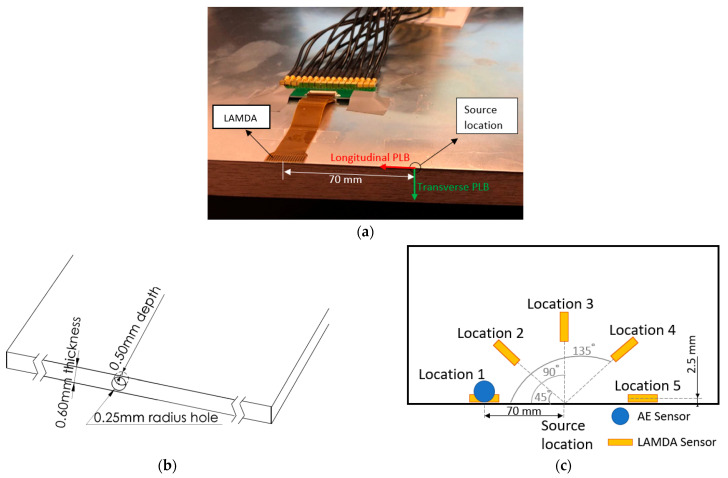
(**a**) PLB test set-up indicating lead-break directions relative to LAMDA orientation; (**b**) schematic showing PLB hole dimensions; (**c**) sensor locations relative to source.

In this experimental study, PLB tests were conducted on the edge of a 400 mm × 400 mm × 0.6 mm aluminium alloy 5005 (Al5005) plate (refer to Figure 1a). PLB tests are usually conducted on the surface of the testing plate, but in practice, it is difficult to conduct PLB tests with a high degree of repeatability [42]. To ensure the PLBs were applied at precisely the same location and in the required direction, a 0.5 mm diameter and 0.5 mm depth reference hole was drilled on the surface of the testing plate (see Figure 1b). A hard-black pencil lead of 0.5 mm diameter was used to generate PLB sources. The pencil lead was inserted into the hole (details in Figure 1b) and broken in the out-of-plane (transverse) and in-plane (longitudinal) directions, as shown in Figure 1a.

Two sensing systems were used to record the AE signals. The first system was a Physical Acoustics Corp micro-SHM system with a 20 kHz–1000 kHz analog filter. This system operates via a threshold trigger and has a sampling rate of 10 MHz. The trigger threshold was set to 40 dB to avoid triggering during the insertion of the pencil lead into the drilled hole. The commercial AE sensor used with the system was a PKWDI sensor from Physical Acoustics Corp. This sensor can only record the response at a single point, which, in this study, was a position 70 mm radially away from the centroid of the source, labelled Location 1 in Figure 1c.

The second system was the LAMDA [40,41], which consists of 16 5 mm × 1 mm piezoelectric elements arranged in a linear array with a 1.27 mm pitch, mounted on a thin flexible polymer carrier. The total length of this sensor is ~20 mm. Signal recordings from LAMDA were acquired using a 16-channel Acousto Ultrasonic Structural health monitoring Array Module^+^ [43] with a 43 μs pre-trigger time, a 0.02 μs sampling rate, and a 50 kHz–5 MHz bandwidth. The output from LAMDA consisted of 16 voltage waveforms from the 16 sensing elements in the array. Further details on the architecture of the LAMDA and the hardware can be found in [40,41,43]. LAMDA sensors were bonded to the plate along the radial direction from the source, as illustrated in Figure 1c, so that the wavenumber–frequency plots obtained from the PLB source could be compared with the theoretical curves obtained from DISPERSE without adjustment for the angle of incidence [40]. Five LAMDA sensors were positioned 70 mm radially from the source and arranged in orientations of 0, 45, 90, 135, and 180 degrees with respect to the plate edge, denoted as locations 1–5, respectively, as shown in Figure 1c. Waveforms at locations 1 to 5 were recorded for longitudinal direction PLB tests, while only waveforms at locations 1, 2, and 3 were recorded for the transverse PLB. The reason for this is that for the transverse PLB test, the system is symmetric, so waveforms at locations 4 and 5 should theoretically be the same as those at locations 1 and 2. The PLB tests were repeated 10 times at each location, and the wavenumber–frequency plots shown in Section 3.2 represent an average taken over the 10 PLB tests.

### 2.2. FEA Analysis

**Figure 2:**
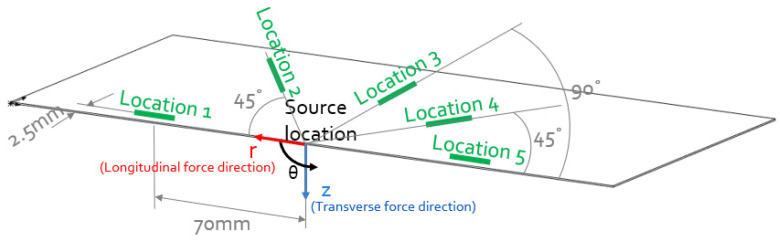
FEA model set up: source position, detecting position, and input force directions.

The arrangement shown in Figure 2 was also modelled using the ANSYS 19.2 Explicit Dynamic FEA package. The aim was to compare measurements of the Lamb wave modes generated in the considered plate using PLBs acting in the longitudinal and transverse directions with corresponding numerical predictions.

The plate considered in this simulation had side dimensions of 280 mm × 140 mm × 0.6 mm, and the detection locations were identical to those used in the corresponding experiment described in the previous section. Cylindrical coordinates were used in the velocity probe when extracting waveform information. The origin of the cylindrical coordinate system was at the source location. The out-of-plane direction is defined as the *z*-axis, the angular direction is defined as the θ-axis, and the radial direction as the r-axis, as shown in Figure 2. The material properties used in this simulation are consistent with those of the plate used in the experiments and for determining the Lamb wave dispersion curves using the DISPERSE software tool [44]; this is, specifically, Young’s modulus of 68 GPa and Poisson’s ratio of 0.33. The size of the hexahedral element was 0.2 mm in both length and width directions, and 0.075 mm in the plate thickness direction. The 0.2 mm length dimension corresponds to ~10 nodes per wavelength for the shortest wavelength of interest (the A0 mode), which is sufficient [45] to accurately decompose all Lamb wave modes below the maximum frequency of interest of 1 MHz. In the plate thickness direction, the 0.075 mm element dimension was determined to be sufficient for an accurate representation of the mode profiles [46].

A linear ramp forcing function was used to simulate the PLB input force [42]. As the dimensions of the hole were small (~20%), relative to the shortest wavelength, the influence of the hole was neglected, and the force was applied directly as a point force at the mid-plane location of the plate. Transverse and longitudinal 0.5 μs linear ramp nodal input force functions were applied and investigated separately (refer to Figure 2).

The time step used in the simulation was 0.05 μs, which corresponds to 20 time steps per cycle at the maximum considered frequency of 1 MHz, satisfying the ANSYS general requirement for accurate results [47]. At each recording location shown in Figure 2, 16 measurement probes were created at locations corresponding to the centre of each of the LAMDA sensing elements.

### 2.3. Theoretical Dispersion Curves

**Figure 3:**
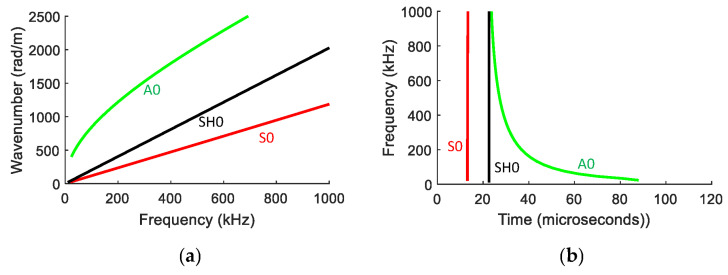
(**a**) Theoretical wavenumber–frequency dispersion curve for 0.6 mm thick aluminium plate. (**b**) Corresponding frequency vs. arrival time 70 mm from the source.

The DISPERSE software tool [44] was used to obtain theoretical dispersion curves (wavenumber–frequency characteristics) for S0, SH0 and A0 modes for the aluminium plate in the frequency range of 0–1000 kHz. These theoretical dispersion curves are shown in Figure 3a. Figure 3b shows the corresponding frequency–time plots, determined from the group velocity dispersion curves and the known source to sensor distance.

### 2.4. Validation of Method

**Figure 4:**
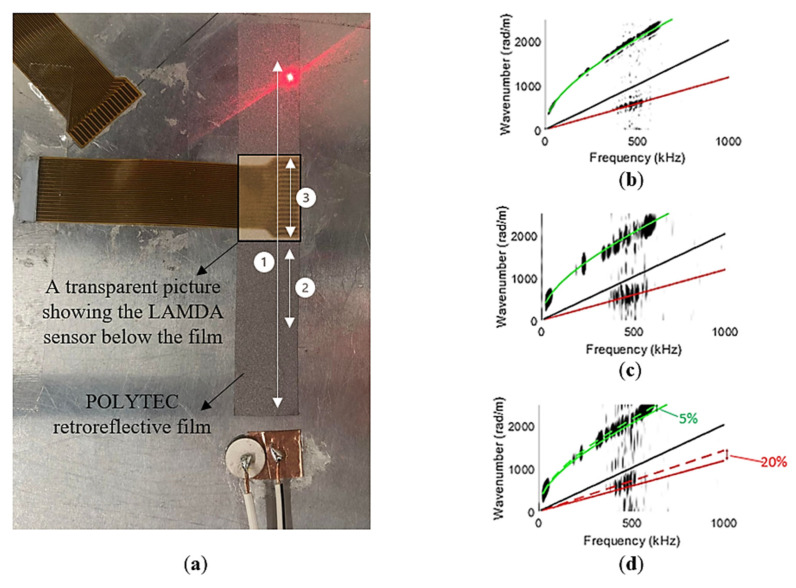
(**a**) Laser scanning routes labelled 1–3. Al5005 plate dispersion curve wavenumber versus frequency (**b**) of route 1 (80 mm); (**c**) route 2 (20 mm); and (**d**) route 3 (20 mm over LAMDA sensor).

To verify the mechanical properties of the aluminium plate, laser vibrometry was used to obtain the Lamb wave dispersion curves experimentally. In addition, in order to explore the impact of the LAMDA sensor on these dispersion curves, laser vibrometry was undertaken on the plate with and without a LAMDA attached. The corresponding dispersion curves were obtained by applying a 2D FFT to the laser vibrometer measurements. A three-dimensional laser vibrometer was used to obtain measurements of the in-plane and out-of-plane displacement components of Lamb waves in the plate. A piezoceramic disc element was used to selectively excite the A0 mode, and a transducer with an angle wedge was used to selectively excite the in-plane S0 mode [48]. To improve the signal-to-noise ratio of these measurements, retroreflective film was used over the scan paths, labelled 1–3 in Figure 4a: route 1 was 80 mm in length, route 2 was 20 mm in length and adjacent to the LAMDA sensor, and route 3 was 20 mm in length and was over the LAMDA sensor.

As can be seen in Figure 4, there is generally good agreement between the theoretically and experimentally obtained dispersion curves for routes 1 and 2. However, for route 3, there is a small discrepancy of ~5% for the A0 mode, and a slightly larger discrepancy of ~20% for the S0 mode, indicating that the LAMDA has an influence on Lamb wave propagation in the plate. Given that the structure and elastic properties of LAMDA are known, it should be possible to compensate for this influence, but this was not required in the present study as the objective is simply to identify constituent modes.

## 3. Results and Discussion

### 3.1. Wavelet Transform Results

**Figure 5:**
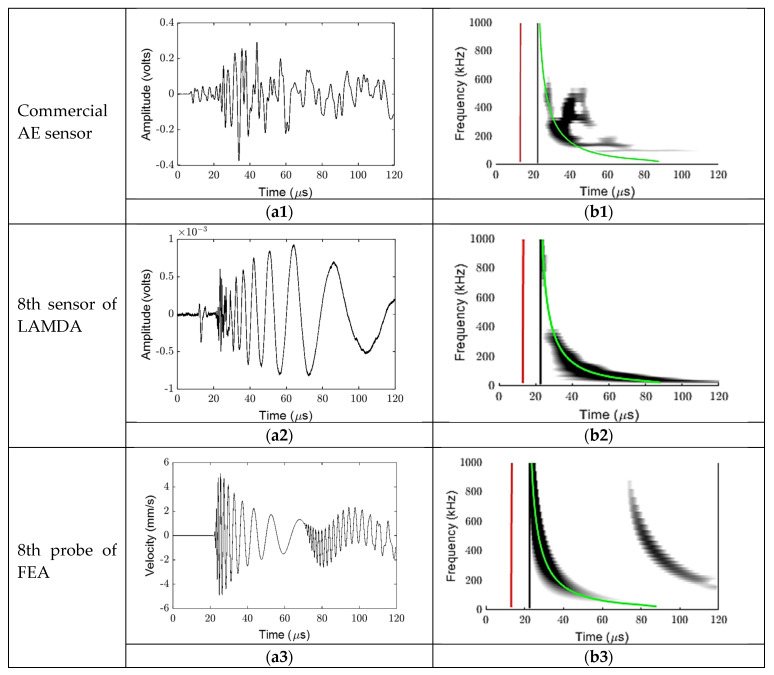
Transverse PLB results. Top row shows waveform (**left**) and corresponding time–frequency plot (**right**) for commercial AE sensor. Middle row as for top row but for LAMDA sensor. Bottom row as for top row but for FEA prediction corresponding to out-of-plane velocity component.

**Figure 6:**
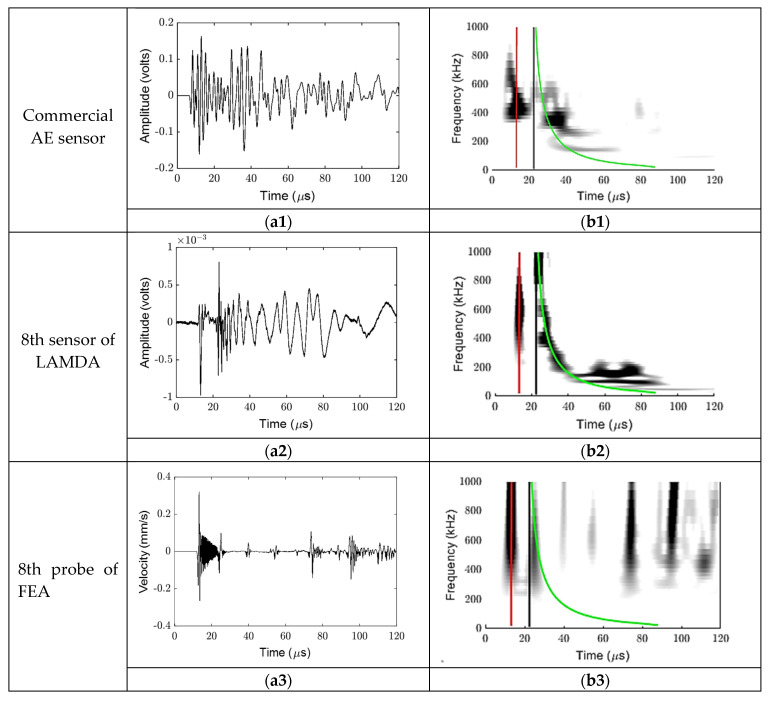
Longitudinal PLB results. Top row shows waveform (**left**) and corresponding time–frequency plot (**right**) for commercial AE sensor. Middle row as for top row but for LAMDA sensor. Bottom row as for top row but for FEA prediction corresponding to radial velocity component.

In Section 3.1, for the purpose of analysing the time–frequency signals, in order to obtain a direct comparison with the waveform acquired from the commercial AE sensor, the LAMDA, and the FEA at the same distance, the waveform acquired from the 8th (middle) sensing element in LAMDA and the FEA probe at location 1 were compared with those acquired from the Commercial AE sensor. The waveforms collected for the transverse PLB using the Commercial AE sensor, the eighth element of LAMDA, and the eighth probe of FEA are shown in Figure 5(a1), Figure 5(a2) and Figure 5(a3), respectively. Time–frequency plots and the corresponding theoretical curves (Figure 3b) are superimposed in Figure 5(b1–b3). The results of the longitudinal PLB tests are presented in Figure 6 with the same arrangement.

For reference, for the FEA generated plots, zero-time corresponds to the time when the force was applied; in addition, signal contributions arriving after 70 μs in Figure 5(b3) and after 30 μs in Figure 6(b3) correspond to reflections from plate edges, and can thus be ignored.

Since measurements from the conventional Commercial AE sensors and LAMDA could not be synchronised with the applied force, the waveform recordings needed to be appropriately time-shifted so that the wave arrival times were consistent with the theoretical group velocities shown in Figure 3b. The required time-shift was determined by ensuring that the experimental frequency–time contour was aligned with the theoretically determined contour. In the case of Figure 5, this contour corresponded to the A0 mode. This process was not as straightforward to apply for the longitudinal PLB results, particularly for the Commercial AE sensor data as it produced a contour markedly different to that of the theoretical result.

From Figure 5, it is clear that the A0 mode is dominant for the transverse PLB orientation, with little evidence of an S0 contribution; for the longitudinal PLB orientation results shown in Figure 6, both A0 and S0 are present in the experimentally obtained measurements; in the simulation results, it is unclear whether the second arrival corresponds to an SH0 mode or an edge wave, which is discussed further in Section 3.2.

The results in Figure 5 and Figure 6 show that the ratio of the S0/A0 modes is dependent on the orientation of the PLB. From the transverse PLB results in Figure 5(b1–b3), the WT coefficient of the S0 mode was extremely small compared to that of the A0 mode. On the other hand, the results obtained with a longitudinal PLB show that the WT coefficient of the S0 mode was about the same as that of the A0 mode, see Figure 6(b1–b3). Therefore, the longitudinal (in-plane) PLB results lead to a higher ratio of the S0/A0 mode compared to the transverse (out-of-plane) PLB. As PLBs on the edge of a plate have been reported to be more similar to the buried AE sources [49], the difference in the modal ratio between the two PLB orientations suggests that LAMDA could potentially be used to distinguish different internal AE sources.

### 3.2. 2D FFT Results

The waveforms obtained from FEA were time-gated to exclude reflected wave contributions before applying a 2D FFT. The length of this time gate was determined from the known Lamb waves group velocities. The wavenumber–frequency plots corresponding to the transverse PLB orientation were normalised with respect to the maximum out-of-plane component amplitude at location 1, while those corresponding to the longitudinal PLB orientation were normalised with respect to the maximum radial component amplitude at location 1. After the normalisation, the wavenumber–frequency plots that corresponded to transverse PLBs from the FEA for the angular component contained no information, as the energy was extremely low, and thus was not shown. Similarly, the wavenumber–frequency plots that corresponded to longitudinal PLBs from the FEA for the out-of-plane component contained no information, and thus were not shown. After this, the results from the FEA were compared with experimental results from LAMDA at all detecting locations.

**Figure 7:**
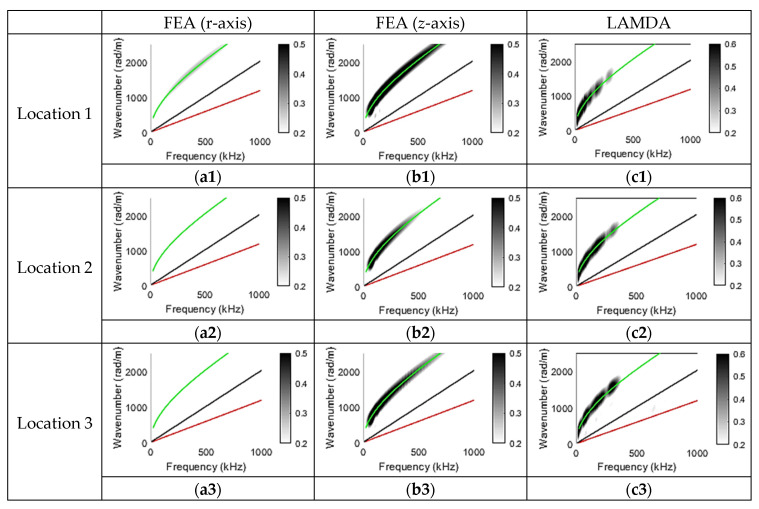
Transverse PLB results. Top row shows wavenumber–frequency plots corresponding to the radial component obtained from FEA, the out-of-plane component obtained from FEA, and from the LAMDA sensor at location 1. Middle row as for top row but for location 2. Bottom row as for top row but for location 3.

The experimental results and the FEA results that correspond to the transverse PLB orientation for sensing locations 1 to 3 are shown in Figure 7. Figure 7(a1–a3) are wavenumber–frequency plots obtained from FEA waveforms for the radial component (r-axis); Figure 7(b1–b3) are wavenumber–frequency plots obtained from FEA for the out-of-plane component (*z*-axis); and Figure 7(c1–c3) are wavenumber–frequency plots obtained from waveforms recorded by LAMDA. When the PLB is applied transversely, the geometry and loading of the plate are symmetric about the centreline, so waveforms at locations 4 and 5 should theoretically be the same as at locations 1 and 2, respectively. Thus, only results from locations 1 to 3 are shown here.

Based on the observation of Figure 7, it can be seen that this forcing configuration leads to the generation of dominantly asymmetric wave modes. The wavenumber–frequency plots that correspond to PLB tests using LAMDA sensors were dominated by A0 mode contributions at all three locations, and the wavenumber–frequency plots from FEA, generated using the out-of-plane component, were dominated by a strong A0 mode contribution, and a very weak A0 mode at location 1, which corresponded to the radial component. The dominance of the A0 mode is consistent with findings from Hamstad’s study [38], where a monopole out-of-plane source near the mid-plane gave rise to the A0 mode only. Additionally, the wavenumber–frequency plots corresponding to the out-of-plane component in the FEA waveform had a slightly higher energy at location 1 than at locations 2 and 3. A possible reason for this could be the presence of an asymmetric edge wave [50]; however, this was not confirmed. The weak A0 mode present in the radial component results shown in Figure 7(a1) is assumed to correspond to the leakage of the asymmetric edge wave.

**Figure 8:**
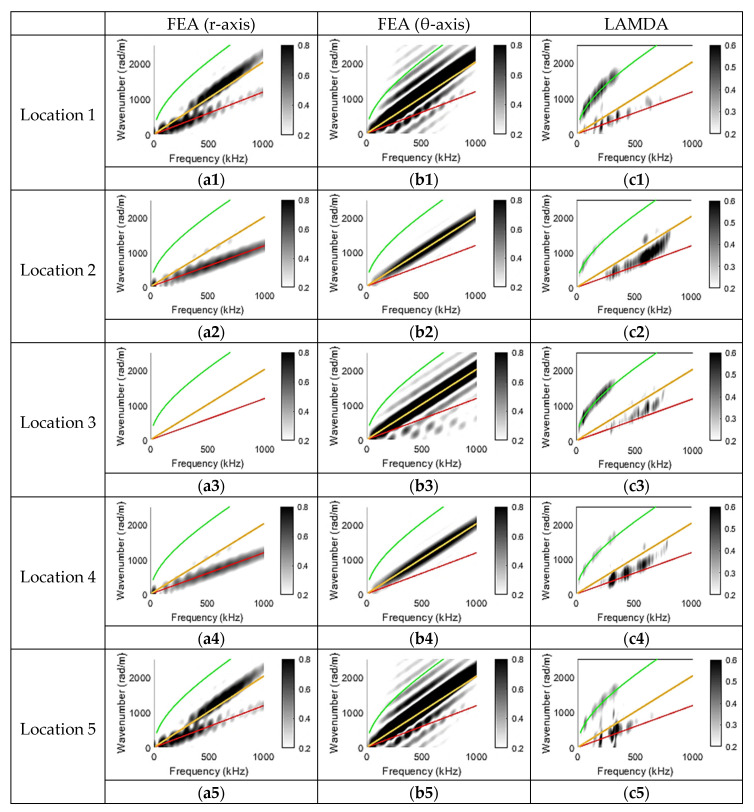
Longitudinal PLB results. Top row shows wavenumber–frequency plots corresponding to the radial component obtained from FEA, the angular component obtained from FEA, and from the LAMDA sensor at location 1. Second row as for top row but for location 2. Third row as for top row but for location 3. Fourth row as for top row but for location 4. Bottom row as for top row but for location 5.

Figure 8(a1–a5) are wavenumber–frequency plots of waveforms from FEA that correspond to the radial component (r-axis); Figure 8(b1–b5) are wavenumber–frequency plots of waveforms from FEA that correspond to the angular component (θ-axis); and Figure 8(c1–c5) are wavenumber–frequency plots of waveforms recorded by LAMDA sensors.

As can be seen in Figure 8(a1–a5), the wavenumber–frequency plots from FEA that correspond to the radial component showed S0 mode content at detecting locations 1, 2, 4, and 5, with almost no energy at location 3. Locations 2 and 4 contained stronger S0 mode contributions above 500 kHz, which was consistent with the corresponding wavenumber–frequency plots obtained from waveforms recorded by LAMDA sensors.

In Figure 8(b1–b5), the wavenumber–frequency plots from FEA waveforms that correspond to the angular component (θ-axis) showed that the SH0 mode appeared to be dominant at locations 2, 3, and 4. However, it was observed that the wave mode contributions at locations 1 and 5 did not align perfectly with the known SH0 mode wavenumber–frequency curve. Moreover, the energy of the suspected SH0 mode at locations 1 and 5 was higher compared to locations 2 and 4. The reason for this was that the symmetrical Rayleigh-like edge wave propagating near the edge of the plate was also captured at locations 1 and 5 [51]. This edge wave is also evident in the radial displacement component at locations 1 and 5, shown in Figure 8(a1,a5). This wave mode could also be confirmed to be an edge wave rather than the SH0 mode from Figure 6(b3), as the velocity of the symmetrical edge wave was expected to be around 90% of the SH0 mode [52].

Locations 1 and 5 and locations 2 and 4 have the same empirical Lamb wave dispersion curves, corresponding to both radial and angular components of FEA and LAMDA sensors. This demonstrates that the longitudinal PLB tests generate a symmetric wave-scattering pattern with respect to the plate centreline.

For the longitudinal PLB tests, both A0 and S0 mode contributions were observed in the wavenumber–frequency plots obtained from the LAMDA sensors at all five locations. Comparing the wavenumber–frequency plots from FEA predictions and the PLB tests, it is observed that the S0 mode observed in Figure 8(c1–c5) was most likely from the radial component of the collected waveform. Furthermore, an A0 mode contribution is observed in the LAMDA wavenumber–frequency plot that is not present in the corresponding FEA predictions. This is because, when conducting the longitudinal PLB test manually, it was almost impossible to keep the force strictly vertical to the plate. As a result, the longitudinal PLB is likely to also have contained a weak transverse component, leading to the excitation of A0. Observing the wavenumber–frequency plots from LAMDA in Figure 7(c1–c3) and Figure 8(c1–c5), it is apparent that a longitudinal PLB results in an additional S0 modal contribution compared to a transverse PLB. For a longitudinal PLB, the S0 modal contribution above 500 kHz is more significant at locations 2 and 4 than at locations 1 and 5.

The presented results indicate that the orientation of a PLB affects the modal composition of the resulting AE. Such modal contributions can be difficult to distinguish using time–frequency analysis only, i.e., synchronising the time–frequency plots with the theoretical group velocity curves may not be sufficient when there are multiple interfering wave modes. By contrast, the wavenumber–frequency decomposition implemented using LAMDA allows contributing wave modes to be determined regardless of time interference. Consequently, LAMDA could provide a useful basis for the modal decomposition of Lamb wave packets generated by other AE sources. Such decomposition may aid in AE source localisation and characterisation.

## 4. Conclusions

This study has demonstrated the ability of a novel multi-sensor array to achieve the modal decomposition of the acoustic emission from PLBs when different orientations of the acoustic source are applied. It was shown that a transverse PLB produces a dominant A0 mode, while a longitudinal PLB produces a combination of A0 and S0 modes. These results are consistent with FEA simulations and previously reported AE studies. The current work includes the investigation of only zero-order wave modes and the application of the LAMDA sensors on isotropic plates. Ultimately, this capability will be used to study the wave mode composition of the AEs generated during fatigue crack propagation in coupons. Future work will investigate the higher-order wave modes that are excited by acoustic sources arising from damage initiation and development. Future work will also include the use of LAMDA to identify the wave modes generated by the different damage mechanisms in composite panels.

## Data Availability

Not applicable.
